# The Effects of Ventriculoperitoneal Shunt on Gait Performance

**DOI:** 10.25122/jml-2019-1004

**Published:** 2019

**Authors:** Dumitru Baltateanu, Ileana Ciobanu, Mihai Berteanu

**Affiliations:** Carol Davila University of Medicine and Pharmacy, Bucharest, Romania; Elias University Emergency Hospital, Bucharest, Romania

**Keywords:** normal pressure hydrocephalus, gait, balance, ventriculoperitoneal shunt

## Abstract

Most studies on patients with normal pressure hydrocephalus (NPH) regard pre-post Tap test and long-term follow-up after shunt surgery. Quantitative and qualitative assessment tools specific to rehabilitation medicine can provide an objective measurement of the benefit of the neurosurgical intervention at 1-month follow-up.

The aim of this retrospective study was to assess the early benefit of the ventriculoperitoneal shunt with low or medium pressure valve on the gait capacity of persons with NPH, one month after surgery.

This is a retrospective study reviewing 19 inpatients with NPH who underwent neurosurgery for ventriculoperitoneal shunt with low or medium pressure valve, one month after a positive result on a tap test, in a 5-year period. The assessments regarding the gait abilities were performed 24 hours before the surgical intervention and one month after surgery. Assessment tools used were: the 3 meters Timed Up and Go Test (TUG), the 10 Meters Walking Test (10MWT) and the Berg Balance Scale.

A positive response to the tap test predicted improvements of gait and balance in patients with NPH after shunt surgery. Best results in regards to gait and balance are achieved when early diagnosis and intervention are performed. Complex comorbidities generate and enhance significant and persistent gait impairment.

## Introduction

Normal-pressure hydrocephalus (NPH) was first described as a diagnosis entity by Hakim and Adams, relatively recent, in 1965. NPH is a potentially reversible neurodegenerative condition and presents by the triad: gait impairment, urinary incontinence and cognitive impairment similar to dementia. The syndrome is associated with the normal pressure of the cerebrospinal fluid and ventricular dilation that is not due to cerebral atrophy [1]. Association of clinical evaluation with revolutionary imagistic investigations such as computed tomography (CT) and especially magnetic resonance imaging (MRI) lead to improved diagnosis and treatment for patients with NPH, this entity being one of the few neurodegenerative conditions that benefit from neurosurgical interventions.

Gait impairments are generally the first sign when an NPH is in progress, and the most visible one [2]. In patients with NPH, gait is characterized by low walking velocity, wide-base walk, small steps and imbalance [3]. A shunt leads to important early improvements in gait parameters, gait being the first and most responsive feature in most cases [4].

Improved ambulation abilities will lead to better personal factors supporting the improvement in the other two aspects: urinary disturbances and cognitive impairment. Thus, a better outcome regarding functioning, the ability to perform usual activities as well as the capacity to participate in everyday life are ensured.

In order to reduce the risk of significant complications, some inclusion criteria for surgical intervention were developed [5]. Patients with NPH responding with improved functioning to a cerebrospinal fluid tap test (TT), also called large-volume lumbar puncture, a commonly used prognostic test requiring removal of 15-5ml of cerebrospinal fluid [6] are referred for neurosurgical intervention and will provide further and sustained functional improvement [7]. TT response is assessed subjectively, by a neurologist or neurosurgeon, but various quantitative objective measurements of the TT response are available and documented as well [5]. The present study aimed to assess the benefits of the neurosurgical intervention for a ventriculoperitoneal shunt with low or medium pressure valve at 1-month follow-up, using internationally-accepted gait and balance assessment tools.

Souza et al. found in their study that gait speed was the most responsive gait parameter after cerebrospinal fluid removal [8]. Different tests such as Timed Up and Go Test (TUG), 10 meters walking speed test (10MWT) and Berg Balance Scale are widely used for assessing the physical functionality of the adults with NPH. TUG time has 0.967 specificity and 0.933 sensitivity for the diagnosis of NPH [9]. A pre-post TT difference of 3.985 seconds in TUG is a predictor of positive response to the surgical intervention in NPH [10]. A TUG time shortened by 5 seconds or more after a TT predicts a 40-60% expectancy of improvement in TUG time at 12 months after shunt surgery, by more than 10 seconds or 5 seconds, respectively [11].

A systematic review and meta-analysis including 25 studies concluded that Berg Balance Scale score (≤50 points), Timed Up and Go times (≥12 seconds), and five times sit-to-stand times (≥12 seconds) are the most evidence-based functional measures to determine the risk of falls [12]. A 2014 literature search indicates that the TUG test is more useful at ruling in rather than ruling out falls in individuals at high risk of falling (TUG >13.5 sec) [13]. Shumway-Cook et al. found in 2000 that the TUG test proves to be an appropriate tool for identifying community-dwelling older persons at fall risk (previous fallers or not).The differences between fallers and non-fallers are consistent in single-task or dual-task performance (motor or cognitive task associated with TUG) [14].

## Materials and Methods

This is a retrospective study, performed over five years (from 01.01.2014 to 31.12.2018). Participants were inpatients of the Neurosurgery Department of Elias University Hospital in Bucharest, who underwent ventriculoperitoneal shunt surgery with a low or medium pressure valve and anti-siphon device. All patients were diagnosed with NPH based on the clinical examination and CT and MRI results. Out of 21 inpatients diagnosed with NPH, 19 participants responded positively to the TT test consisting in a lumbar puncture and evacuation of 20-30 ml of cerebrospinal fluid. The surgical intervention was performed under general anesthesia with orotracheal intubation, four weeks after TT testing. The patients were discharged from the hospital in 3-5 days after surgery and reexamined clinically and imagistically (using CT scanning) one month after surgery.

The functional assessment was performed in the Rehabilitation Medicine Department of Elias University Hospital in Bucharest and included the Timed Up and Go test – 3 meters version (TUG) (3 times means) [11], the 10m walking test at the self-chosen comfortable speed [15] and the Berg Balance Scale [16]. The study compares 24h pre-surgery assessment results with one-month follow-up after surgery.

## Results

Nineteen patients responding positively to the tat test were included: 7 females and 12 males, 17 from urban communities, 2 from rural environments, with an average age of 69 years and 6 months (60-77 years old).

Most of the participants presented comorbidities: 14 with high blood pressure (HBP), 10 with diabetes mellitus type 2 (DM2), 5 with Parkinson’s disease (PD), 2 with atheromatosis, one with thrombosis, 2 with heart failure (HF), 2 with glaucoma, one with post-stroke tetraparesis and one with hypothyroidism.

The time from the onset of symptomatology and the diagnosis varied between 1 month and 12 months, the average being five months.

**Figure 1:**
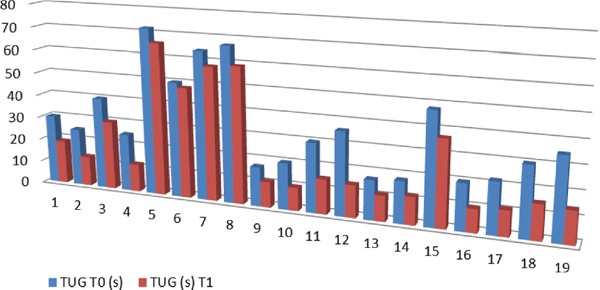
Initial (T0) and follow-up (T1) TUG times (s)

On average, the TUG time in the current study improved by 36.48% (from 37.68 s to 24.42 s). Two participants improved by more than 20 s their TUG time (from 36 s and 35 s to 14 s), ten improved their TUG time by 10-19 s, six improved their TUG time with 6-9 s and only one improved the TUG time by only 2 seconds (from 50 s to 48 s). The longest TUG time improved with 8.3%, from 72 s to 66 s, in a patient with complex comorbidities (DM2, HBP and PD) (Figure 1).

**Figure 2:**
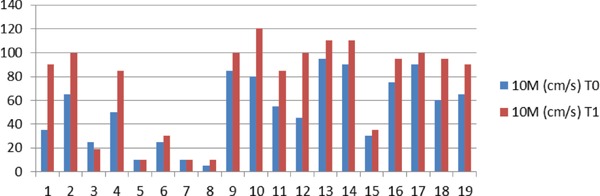
10MWT speed at T0 (initial evaluation) and T1 (follow-up)

The average speed in 10 MWT increased by 216.3 mm/s, with a percentage of 45.4, from 523 mm/s to 734 mm/s (Figure 2). The lowest initial speeds have improved as well. Two patients presenting initially low-speed gait (100 mm/s) that needed significant assistance showed no improvement in gait speed but showed some improvements in TUG time and Berg score (9.36% and 8% in TUG, 8 and 6 points on the Berg scale).

**Figure 3:**
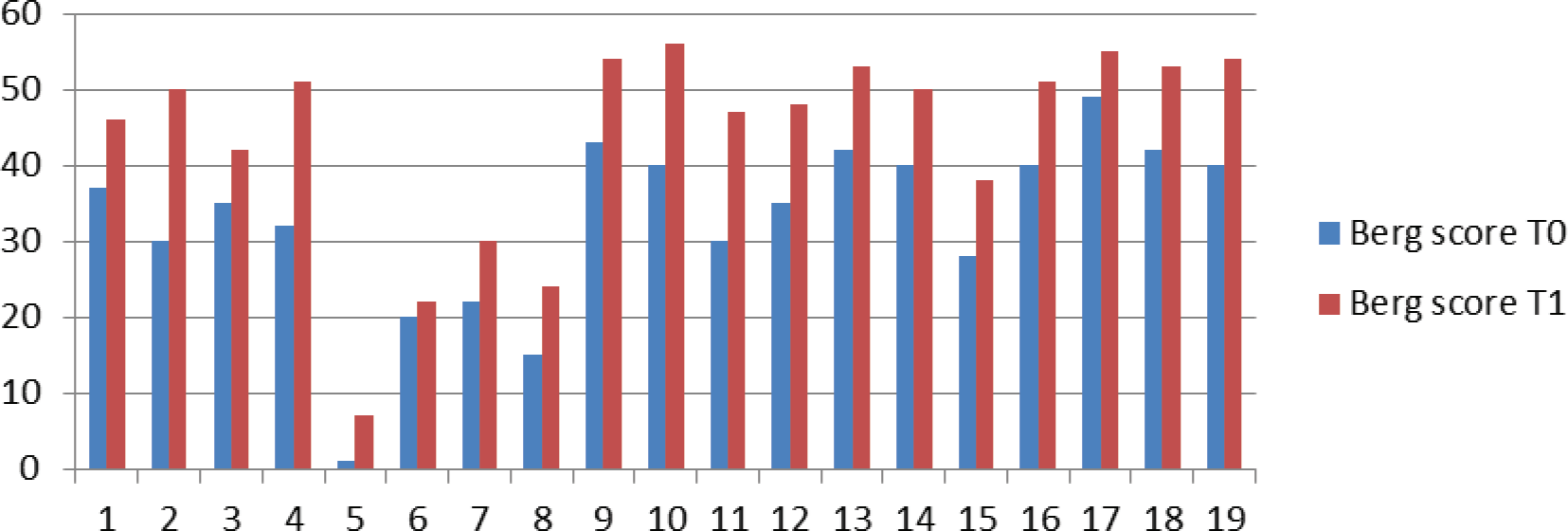
Scores on the Berg Balance Scale at T0 (initial evaluation) and T1 (follow-up).

The Berg scale score improved with 11.57 points, on average. The best improvements were achieved by patients with early diagnosis and intervention: 1-4 months from the debut (10-20 points increase). 12 participants showed an improvement in the Berg score of more than 10 points (Figure 3).

## Discussion

The participants in this retrospective study presented with the classical triad of symptoms, with different degrees of gait impairments. Most of the participants presented with significant comorbidities; 5 had Parkinson’s disease, which is frequently found in patients with NPH as reported by Ishii et al. [17]. Diabetes mellitus, high blood pressure and cerebrovascular disease are described as highly associated with NPH by Israelson et al., as confirmed by our study [18].

In the present study, 18 out of the 19 participants were referred to neurosurgery after positively responding to the tap test and showed significant clinical improvement in the recorded parameters one month after the surgical intervention, being in accordance with authors reporting that the tap test has a predictive value for improving gait between 72 and 100% and a low rate of complications after surgery [19].

All the participants in this retrospective study had initial TUG times over 16 seconds (16.5 – 80 s), confirming the diagnosis in terms of functionality as well. Mendes et al. and other authors used a cut-off of 16.5 seconds for TUG time as diagnostic criteria for NPH [9].

The participants showed a 36% improvement of TUG time at follow-up, 18 of them showing TUG times reduced by more than 6 seconds and better functioning, in accordance with Billek and Jackon’s discovery that a TUG time reduced by 2.5 s after an intervention can be considered a clinical improvement [20]. Huang et al. indicate an interval of 3.5 seconds as being the minimal detectable change in TUG for patients with Parkinson’s disease; therefore, the changes achieved in the present study are essential regarding patients with PD as well [21]. Thirteen participants presented at follow-up with an improvement in TUG time greater than 30%, indicating an improvement of all tasks involved in the TUG test, as Carroni et al. also indicated [22].

In our study, the average gait velocity change was found to be 216 mm/s. Only two participants had no improvement regarding gait speed, and the lowest improvement (50 mm/s) was recorded for three of the participants. As Perrera et al. reported, the smallest meaningful change estimates for gait speed in geriatric patients ranged from 50 mm/s to 130 mm/s and represented a substantial change [23]. Our results indicate a clinical improvement as well.

In accordance with the scores achieved on the Berg Balance Scale, 3 participants of the present study pertaining to the category of whellchair-bound patients (0-20 points) were categorized as individuals that can walk with assistance, and 7 participants from the category of individuals walking with assistance (21-40 points) upgraded to the category of independent individuals (41-56 points) [24].

At the initial evaluation, all the participants in the present study had TUG times greater than 14 s. During follow-up, 7 participants had TUG times lower than 13.5 s, presenting a much more reduced risk of falling.

In the present study, 8 participants showed score improvement in the initial category on the Berg scale. The participants presenting the most severe gait and balance initial impairment (due to a cumulus of neurological and metabolic conditions) achieved 6 points on the Berg scale (from 0 to 7). Regarding the participants that initially presented a risk of falling, 9 participants upgraded to the category of non-fallers (more than 42 points, despite the history of falls), in accordance with the score cut given by Shumway- Cook in 1997, with 91% sensitivity and 82% specificity [14].

The risk of falling gives an essential indication regarding the elderly’s ability to live independently in society and participate in family and community life, as community ambulation involves dual-tasking most of the time [25]. Best results in gait speed seem to be obtained when interventions are performed as early as possible after the onset of NPH. Souza et al. reported an average improvement of 130 mm/s (45.3 s at T0 and 35.2 s at T1 for 20 m walking, pre- and post-tap test) at 22.9 months after onset, on average [8]. The improvement in gait speed in the present study is approximately 216 mm/s (45.4%). At the 12-month follow-up after NPH debut, even the lowest performers presented an improvement of 11% and 16% in gait speed, respectively.

The authors of this study found no paper reporting similar assessment timetable. Most authors offer results of functional assessments performed pre- and post-tap test or pre-tap test and 3-month, 6-month or 12-month follow-up after surgical interventions.

The low number of participants and the wide array of comorbidities and gait impairments made impossible any stratification and statistical data processing. The study results are a series of cases, all showing improvements in different aspects considered.

A positive response to the tap test predicted improvements of ambulation abilities in patients with NPH after shunt surgery, NPH being one of the few neurodegenerative conditions with benefits from neurosurgical interventions.

Ventriculoperitoneal shunt with low or medium pressure valve proves to be beneficial in terms of improvement of gait parameters and increases the independent functioning of the patients.

In persons with NPH, best results regarding gait and balance are achieved when early diagnosis and intervention are performed.

## Conflict of Interest

The authors confirm that there are no conflicts of interest.
